# Hypertension-related Knowledge and Its Relationship with Blood Pressure Control in Hypertensive Patients Visiting a Semi-private Tertiary-care Charity Hospital in Karachi, Pakistan

**DOI:** 10.7759/cureus.5986

**Published:** 2019-10-24

**Authors:** Mohammad Khurram Nadeem, Anum Mari, Sundus Iftikhar, Adeel Khatri, Tooba Sarwar, Muhammad Junaid Patel

**Affiliations:** 1 Cardiology/Internal Medicine, The Indus Hospital, Karachi, PAK; 2 Internal Medicine, Ziauddin Medical University, Karachi, PAK; 3 Statistics, Indus Hospital Research Center, The Indus Hospital, Karachi, PAK; 4 Emergency Medicine, Patel Hospital, Karachi, PAK; 5 Internal Medicine, The Indus Hospital, Karachi, PAK

**Keywords:** hypertension, knowledge, blood pressure control

## Abstract

Introduction

Hypertension is one of the leading causes of mortality worldwide. Fifty-four percent of strokes and forty-seven percent of cardiovascular deaths are caused by suboptimal control of blood pressure. Economically developing countries like Pakistan are heavily burdened with an ever-rising epidemic of cardiovascular disease and stroke morbidity and mortality. Therefore, urgent steps are required to treat, as well as modify, risk factors for cardiovascular disease, including hypertension.

Purpose

The objective of this study was to ascertain the knowledge of hypertension and other sociodemographic variables and their impact on controlling blood pressures in the hypertensive population belonging to the low socioeconomic strata.

Methods

This cross-sectional study was conducted in the general medicine and cardiology outpatient clinics of a tertiary care charity hospital. Three-hundred thirty-five hypertensive patients of age >24 years were selected and informed consent was obtained. Hypertension-related knowledge was assessed using the Modified "Hypertensive Knowledge-Level Scale (HK-LS)" via a 15-20 min interview. Secondary variables in the questionnaire included social demographics, medical history, and assessment of body mass index (BMI) and blood pressure average values, which were measured during the interview. Knowledge was recorded based on the 33-point modified HK-LS scale, whereas secondary variables were not counted toward the assessment of knowledge.

Results

The frequencies of low, moderate, and high levels of hypertension-related knowledge were recorded as 2.1%, 79.4%, and 62%, respectively. Among 335 patients, (57.3%) were male, the mean age was 52.5 ± 11.5 years, and 63.6% were professionally active. Median systolic blood pressure (SBP) and diastolic blood pressure (DBP) in hypertensive patients were 140 and 86 mmHg, respectively. Sixty-nine percent of patients reported existing comorbidities, 54% had diabetes, 20.7% had cardiovascular disease, and 24% reported renal disease. No significant association was observed between the levels of knowledge of hypertension and gender, blood pressure (BP) status, professional activity, and age groups (p=0.877, p=0.863, p=0.125, and p=0.400, respectively).

Conclusion

The majority had adequate knowledge of hypertension but only 64.8% had controlled BP status. This depicts not a lack of knowledge and awareness but rather a lack of prevention of risk factors related to hypertension. Thus, further studies are advised to look into the preventive strategies employed by patients to control their BP and assess their effectiveness.

## Introduction

Non-communicable diseases (NCDs) are, by far, the leading cause of death worldwide. The major NCDs responsible for these deaths included cardiovascular diseases, causing about 17.9 million deaths that account for 44% of all NCD deaths and 31% of all global deaths in the year 2016 [1]. Cardiovascular diseases are also now no longer restricted to economically developed countries but are considered endemic worldwide. Fifty-four percent of strokes, 47% of ischaemic heart disease, 75% of hypertensive disease, and 25% of other cardiovascular diseases worldwide begin secondary to indolently developing high blood pressure (BP) [2]. While there has been an improvement in age‐standardized hypertension (HTN) control from 1999 through 2016, however, the absolute burden of HTN has consistently increased [3].

The percentage of the population with controlled blood pressure (BP) status also varies considerably among countries, for example, in developed nations like Canada, the percentage of population with controlled BP is 65% in contrast to the 6% found in Pakistan [4]. In a metropolitan city like Karachi, HTN prevalence was reported to be 12.2% in the 18-34 years age group and approximately 50% in the 55-75 years age group [5]. Economically developing countries like Pakistan are thus found to be at a greater risk of mortality related to HTN [6]. The inadequate control of hypertension can be accredited to demographic characteristics, health beliefs, other chronic diseases, but, more importantly, lack of awareness and knowledge of high blood pressure [7]. Awareness regarding high blood pressure and knowledge of hypertension control is either limited or non-existent in the Pakistani population. In a study conducted in Quetta, it was found that only 0.8% had adequate knowledge of hypertension. When adherence to treatment was assessed, 64.7% of the patients were found to be poorly adherent and no one was found to be a good adherent [8].

While it is difficult to modify the demographic and behavioral patterns and norms and socioeconomic status of the hypertensive population, increasing their knowledge through educational interventions on treatment, however, can positively influence patients’ beliefs about medicines and help vulnerable countries like Pakistan control the burden of the disease of HTN and, through it, many of the deaths due to cardiovascular and other related causes. Earlier studies, which are very few in number, depict that hypertension-related knowledge is extremely poor in the Pakistani hypertensive population and that they were mainly focused on private tertiary care hospitals, which are mostly accessed by the middle-class and elite populations [8]. In an effort to develop preventive strategies for cardiovascular diseases stemming from high blood pressure in the population belonging to the low socioeconomic strata, this study was conducted in a tertiary care charity hospital, which caters to patients from a low socioeconomic status to assess their knowledge status regarding their disease and to assess the relation between knowledge related to hypertension and its impact on blood pressure control.

## Materials and methods

It was a cross-sectional study conducted at a tertiary-care charity hospital in Karachi, The Indus Hospital. Patients with hypertension for one or more years and taking antihypertensive medications were selected for the study from the general medicine and cardiology outpatient departments (OPDs). The age criterion was set as more than 24 years of age and diagnosis of hypertension was confirmed via a thorough history. Patients presenting with hypertension for the first time were excluded and so were patients with secondary hypertension and females with gestational hypertension. Ethical approval was granted by the Indus research department. Interviews were conducted by medical trainees and third-year medical students. Patients were selected as per the inclusion criteria after informed consent was taken.

Patients presenting to the outpatient general medicine and cardiology clinics at The Indus Hospital were considered for this study. The modified “hypertensive knowledge level scale (HK-LS)” scale was employed to assess the knowledge. Besides the 33-item-based HK-LS, further questions were introduced in the interview to assess the demographic characteristics of the patients, medical history, height, weight, substance use, information about the duration of hypertension, systolic and diastolic blood pressure readings, comorbidities, and intake of antihypertensive medications. Additional questions went on to address drug compliance, diet and lifestyle modifications, and the complications of hypertension. The questionnaire was designed after extensive review by experts in the field of hypertension and research methodologies. Each correct answer was given one point while an incorrect answer was allocated zero. The minimum score was zero and the maximum score was 33. Only the 33 questions falling under the modified HK-LS were counted toward the score; the rest of the questions were not marked. Each interview took about 15-20 min to complete. Blood pressure was recorded with the patients sitting in a relaxed position for 15 minutes; the average of three readings was noted from each arm alternatively, using the manual sphygmomanometer. The readings were recorded by the research staff in the provided space on the interview proforma. The interviewer filled in the form, noting the demographic variable, ethnicity, formal education ascertained by the total number of schooling years, occupation, history of hypertension and its management, comorbidities (myocardial infarction, congestive heart failure, cerebrovascular accident, diabetes, renal disease, etc.), substance abuse, height and weight taken, and, finally, the HK-LS answers were noted down with the last seven questions contributing to determining the basic knowledge of the patient regarding hypertension.

## Results

**Figure 1:**
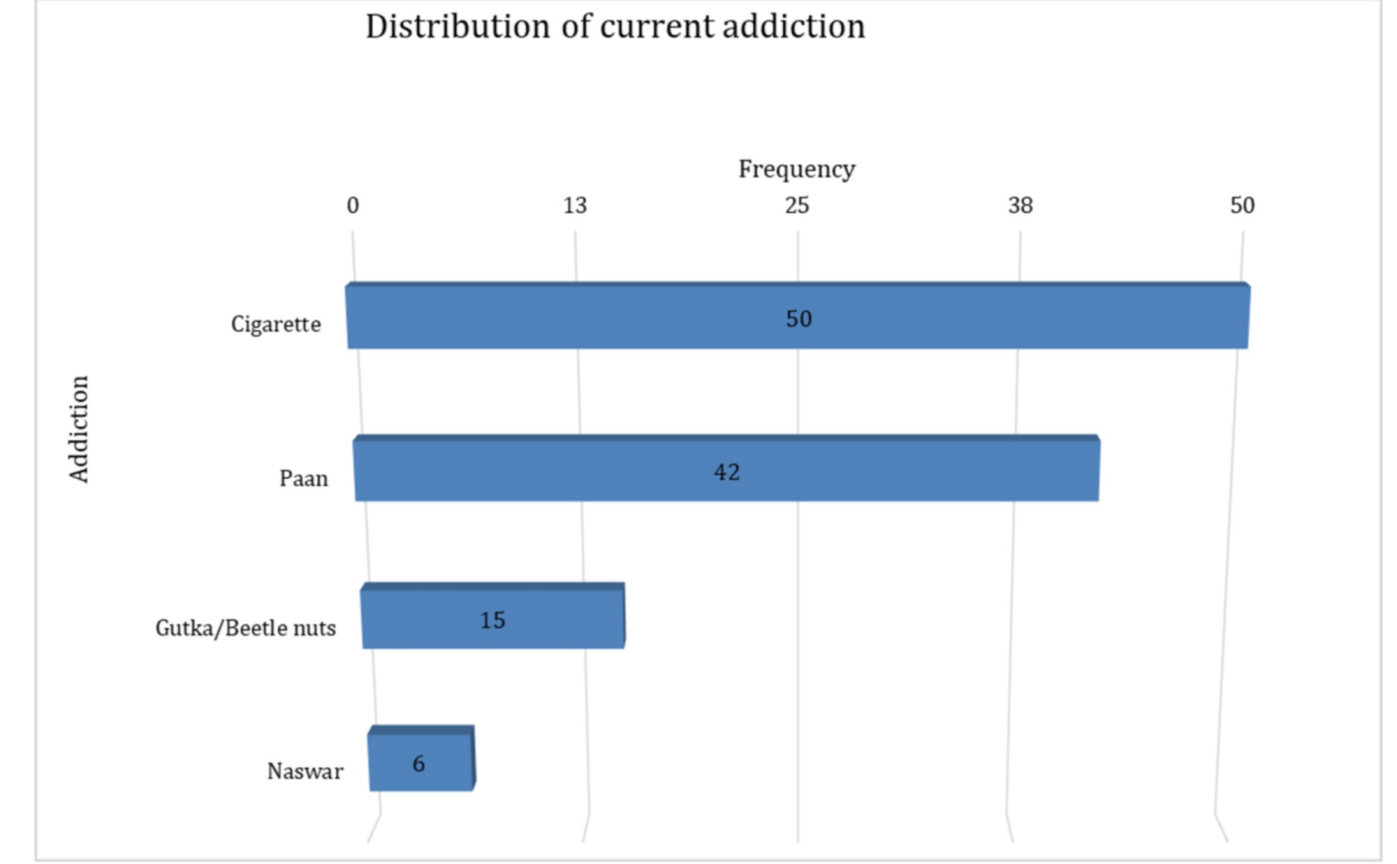
Distribution of current addiction

A total of 335 patients were included in the study, of which more than half were females 192 (57.3%). The mean±SD age of patients was 52.5±11.5 years. The majority of the patients were Urdu speaking 144 (43%). It was observed that 213 (63.6%) hypertensive patients were not working for a living. The median SBP and DBP in hypertensive patients were 140 and 86 mmHg, respectively (interquartile range (IQR): 130-146 and 80-90, respectively. The median duration during which the patients were suffering from hypertension was five years (IQR: 3-9), and they were taking medications for the same for a median of four years (IQR: 2- 8), postulating that patients started medication one year after diagnosis. In 225 (67.2%) patients, there was a family history of hypertension while 139 (41.5%) patients had a family history of cardiovascular disease. A total of 111 (34%) patients were addicted to some kind of substance in the past while 104 (31.8%) patients were found to be currently addicted to paan, beetle nuts, cigarettes, and naswar (Figure 1).

**Figure 2:**
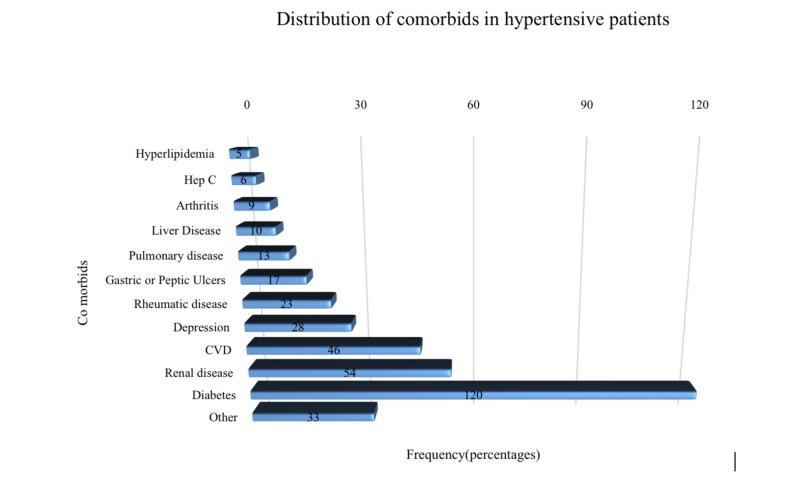
Cormobids in hypertensive patients

The maximum number of patients had diabetes mellitus as comorbidity, 120 (54.1%); followed by renal problems, 54 (24.3%); heart problems 46 (20.7%); and others (Figure 2).

When asked questions related to knowledge of hypertension, 266 (79.4%) patients demonstrated moderate knowledge, 62 (18.5%) showed high knowledge, while 7 (2.1%) patients had low knowledge. After classifying them in two groups on 50% cut-off knowledge, 317 (94.6%) had high knowledge and 18 (5.4%) had low knowledge.

The median age of males was 56 years as compared to females, whose median age was 50 (IQR: 45.8-65 vs 42 -58) years, though males are older than females and the p-value was statistically significant (p<0.0001, Table 1). However, we did not find any age differences in the groups for professional activity, BP status, and knowledge of hypertension, though the p-value was not statistically significant (p=0.080, p=0.629, and p=0.708, respectively, Table 1).

**Table 1:** Characteristics of patient population and association between median HKLS score and groups of gender, professional activity, and BP status BP: blood pressure; BMI: body mass index; HKLS: hypertension knowledge level scale

Age (years)					
	N	Mean±SD	Min-Max	Median (IQR)	P-value
Gender					
Male	142	55.6±12.1	29-82	56(45.8-65)	0.000^** ⱡ^
Female	187	50.2±10.4	25-80	50(42-58)	
Professional activity					
Active	105	51.1±10.1	29-80	50(44-60)	0.080^ ⱡ^
Nonactive	211	53.6±11.6	30-82	52(45-62)	
BP status					
Controlled	213	52.8±11.9	25-82	52(43-62)	0.629^ ⱡ^
Uncontrolled	116	52.2±10.7	30-77	50(45-60)	
Hypertension knowledge					
Low knowledge	18	51.6±12.3	32-73	49(42.3-62.3)	0.708^Ɨ^
High knowledge	311	52.6±11.4	25-82	51(44-61)	
BMI					
Gender					
Male	136	26.5±4.2	15.1-39.8	25.5(24.4-28)	0.000^ **ⱡ^
Female	178	28.9±6.4	12.2-48.1	27.3(24.8-32.1)	
Professional activity					
Active	102	31±8.1	12.2-41.4	29.8(25.8-38)	0.017^** ⱡ^
Nonactive	198	26.6±4	19.2-41.5	25.5(24.6-27.4)	
Hypertension knowledge					
Low knowledge	16	25.1±4.3	17-35.6	25.2(22.3-26.2)	0.043^*ⱡ^
High knowledge	298	28.0±5.7	12.2-12.2	26.0(24.7-30.8)	
BP status					
Controlled	205	27.5±5.6	12.2-46.8	25.6(24.4-30.3)	0.067^ ⱡ^
Uncontrolled	109	28.5±5.9	13-48.1	26.4(25-30.9)	
HKLS score					
Gender					
Male	143	16±3	8-22	16(14-18)	0.620^ ⱡ^
Female	192	15.8±3	4-22	16(14-18)	
Professional activity					
Active	106	16.2±2.9	8-21	16(14-19)	0.245^ ⱡ^
Nonactive	213	15.7±3	4-22	16(14-18)	
BP status					
Controlled	217	15.7±2.9	4-22	16(14-18)	0.306^ ⱡ^
Uncontrolled	118	16.1±3	8-22	16(14-18)	
*p-value<0.05, ** p-value <0.001, ⱡ Mann Whitney U test, Ɨ independent sample t-test					

We observed female patients had a higher BMI as compared to male patients (median 27.3 vs 25.5, p<0.0001, Table 1). Moreover, those patients who were professionally active had a higher BMI than those who were not professionally active (median: 29.8 vs 25.5 P=0.017, Table 1). Unexpectedly, patients with a higher BMI were noted to have a higher knowledge of hypertension. No significant difference in BMI was seen between the controlled and uncontrolled BP groups (p=0.067, Table 1).

Similarly, no significant difference was observed in the median HKLS score between the groups of gender, professional activity, and BP status (p=0.620, p=0.245, and p=0.306, respectively, Table 1).

No significant association was observed between the levels of knowledge of hypertension and gender, BP status, professional activity, and age groups (p=0.877, p=0.863, p=0.125, and p=0.400, respectively, Table 2).

**Table 2:** Association between levels of knowledge of hypertension and the gender, BP status, professional activity, and age groups BP: blood pressure; BMI: body mass index

	Hypertension knowledge level scale			p-value
	Low n (%)	High n (%)	Total n (%)	
Gender				
Male	8(5.6)	135(94.4)	143(100)	0.877^Ɨ^
Female	10(5.2)	182(94.8)	192(100)	
Total	18(5.4)	317(94.6)	335(100)	
BP status				
Controlled	12(66.7)	205(64.7)	217(64.8)	0.863^Ɨ^
Uncontrolled	6(33.3)	112(35.3)	118(35.2)	
Total	18(100)	317(100)	335(100)	
Professional activity				
Active	3(16.7)	103(34.2)	106(33.2)	0.125^Ɨ^
Non active	15(83.3)	198(65.8)	213(66.8)	
Total	18(100)	301(100)	319(100)	
Age group				
≤40	4(22.2)	44(14.1)	48(14.6)	0.400^ꬷ^
41-50	6(33.3)	107(34.4)	113(34.3)	
51-60	2(11.1)	81(26)	83(25.2)	
≥61	6(33.3)	79(25.4)	85(25.8)	
Total	18(100)	311(100)	329(100)	
*p-value<0.05, ** p-value <0.001, Ɨ Chi-square test, ꬷ Fisher exact test				

## Discussion

Patient’s knowledge pertaining to hypertension, its risk factors, and its prevention and control strategies are all important factors in achieving blood pressure control [9]. Our study results showed more than 65% of patients had a significant family history of hypertension and about 41.5% had a positive family history of cardiovascular illness. Fifty-nine point four percent were addicted to pan, beetle nuts, and other forms of mouth intoxicants. Forty-eight percent were current smokers, 54% had diabetes mellitus as a comorbid, 24% had renal disease, and 20% had cardiovascular disease. Similar to our study, a study conducted in Europe reported 24.3% hypertensives with type 2 diabetes, 48.7% with hyperlipidemia, 24.2% had coronary artery disease (CAD) while 28.1% had a family history of CAD and 37.9% were current/former smokers [10]. A similar hypertensive profile may be indicative of an emerging hypertensive crisis in developing nations similar to that already found in developed nations on account of increasing westernization. In the present study, female participants were 57% of the total, a finding in contrast with other studies where male dominance was seen [10-15]. We also found a significant difference in presenting age and gender. Males presented later in age than females, which may be because our study comprised a sample of known hypertensives and women are documented to have a greater sense of health responsibility than men, thus presenting early to the healthcare center [16]. Females were found to have a significantly higher BMI than males, which can be attributed to them being less active professionally. High BMI was associated with a greater degree of knowledge of hypertension, signifying that high BMI individuals had adequate knowledge but their knowledge was not put to use to implement preventive strategies to control BMI and hence their raised blood pressure. Professionally active individuals had a higher BMI than those inactive, which could imply that their professional timings did not allow them to get enough time for taking care of their physical health.

According to our study, 64.8% had controlled BP readings in contrast to less than 30% of hypertensive patients in the United States, based on the Joint National Committee on Prevention, Detection, Evaluation, and Treatment of High Blood Pressure (JNC) VI criteria. Another study found controlled blood pressure in only 3% of all hypertensive patients; this could be attributed to our sample population being those visiting a tertiary hospital while their sample was of the general population [17-18]. An overwhelming majority (80.9%) people in our study affirmed that being symptomless denotes normal blood pressure, 46.6% individuals thought they must take their medication only when they feel ill, regardless of their BP measurement, a finding similar to a previous study in Nigeria where (89%) of the hypertensives, of all educational backgrounds, were unaware of the symptomless nature of the disease [11]. Our study results showed that although 92.5 % agreed that not treating HTN could lead to dangerous complications, such as stroke, blindness, kidney failure, and death, almost half thought blood pressure medications could be taken in a manner that makes them feel good and 49.9% thought medication alone was enough for controlling high blood pressure and thus there was no need to modify their lifestyle. This is consistent with a study conducted in Africa where fewer practiced lifestyle modifications [11]. According to a study, less than 75% of people under medical therapy for hypertension continue using medicines after six months of initiation, and this is associated with an increased risk of hospitalization for cardiovascular problems [19]. Similar to this, our study results depicted a high percentage (52.2%) thought medications were required only when patients ‘feel’ ill. Within the domain of knowledge about hypertension, a previous study found the highest level of knowledge was by lifestyle, then by complications, and then medical treatment whereas in our study, higher knowledge was about complications (84%-94%), medical compliance (71%), and then lifestyle (49.9%) changes, which indicates the necessity of physicians to counsel patients because it is well-known that lifestyle is a single important factor for most chronic diseases, e.g. diabetes mellitus, hypertension [7].

Hypertensive patients' basic knowledge and awareness of risk factors of hypertension and its complications is considerably good, as 79.4% of patients had moderate knowledge of hypertension, while on the 50% cut-off, 94.6% had high knowledge. Our observation is in accordance with the findings of previous studies who found hypertensives demonstrated knowledge, positive perception, and attitude to the treatment of hypertension [20-21]. A moderate amount of public knowledge about hypertension is important because it serves to support health professionals’ efforts in the prevention and treatment of high blood pressure [22]. Our study is in contradiction to the results of other studies that reported limited knowledge of hypertension [23]. Knowledge gaps have also been reported about the risk factors of hypertension in the Gulf region [24]. The characteristics of the study population in those studies were different than in our study, as participants belonged to rural areas and had lower socioeconomic and educational backgrounds when compared to our study population. We found no significant association of knowledge with high blood pressure in contrast to the previous study [4]. We found 35% had uncontrolled BP status and 70.5% did not know the basic definition of hypertension; most patients perceived SBP to be a more important indicator than DBP, 41.8 % were unable to report the link of diastolic blood pressure in indicating high BP while a high percentage (77%) understood SBP levels to be an indicator, which may be the reason for a high percentage of good BP (64.8%), signifying that proper basic knowledge was either not imparted at all or in a conducive way that could be remembered easily by patients. This clearly indicates that there is a lack of transfer of knowledge by physicians to patients. The role of physicians has already been appraised in previous studies [25]. Our study results showed no association between controlled BP status and hypertension knowledge, in contrast with previous studies that showed an association between hypertension knowledge and compliance with treatment in hypertensive patients [4].

Previous studies have concluded that professional activity relates to low knowledge scores in hypertensive patients. We found there was no significant association in the median HKLS score between groups of gender, negative associations with professional activity, and blood pressure status. Although it was found in a few studies that the level of knowledge about hypertension is affected by gender, women showing a higher level of knowledge with regards to the definition and complication of HTN, in some others, it was found that men have higher knowledge [26-27]. Our study couldn't find any significant association between gender groups and the level of HKLS scores. A study also reported a significant association of knowledge regarding hypertension with uncontrolled BP status and ethnicity, in contrast to our study where we couldn't find any significant association [5]. BMI, like age, was directly associated with knowledge of hypertension in our study, similar to a previous study [28].

This study is a single tertiary hospital-centered study focusing on a full analysis of knowledge regarding hypertension of the hypertensive population on medications for more than one year. It found a level of knowledge of specific aspects of hypertension, by taking care of which hypertensives may improve their blood pressure control. The questionnaire used for knowledge assessment was a validated HKLS. Despite the comprehensive nature of this survey, a few limitations of this study are as follows. First, our study was confined to a single center in the urban area, therefore, results cannot be generalized to the rural population. Second, the white-coat effect was not taken into account, which could have possibly resulted in the difference in the BP levels of visiting patients. No monitoring regarding the quality assurance of BP measurements was carried out, therefore, it is not known to what degree doctors complied with the rules. Our study did not take into account compliance with drug therapy by the patient. We obtained no data on drug classes, doses used, and the dosing schedule, to carefully investigate their adherence to medical treatment. Our sample was limited to hypertensives so it cannot be generalized to the whole population, those who have it but are yet not diagnosed, and those who are likely to have it in their future. Their knowledge is also important. We did not analyze the sociodemographic and educational variables to find their association with clinical variables levels and knowledge of hypertension.

## Conclusions

It is concluded from our study results that hypertensive patients had adequate knowledge of hypertension except in the areas of lifestyle modification; however, despite their adequate knowledge, many patients did not have controlled BP status. This depicts not a lack of knowledge and awareness but rather a lack of prevention of risk factors related to hypertension. Effort on the part of physicians is needed to increase the level of knowledge about lifestyle changes, the basic concept of hypertension, and drug compliance by educating and motivating patients and public enlightenment in a conducive manner, e.g. by pamphlets or posters about their target blood pressure according to age, family history, and comorbids, and reinforce the need for patient’s compliance with antihypertensives for life, when required, in order to control the disease and decrease the associated morbidity and mortality. Alternatively, educating patient groups could be an intervention to be tested to improve knowledge of these aspects. We recommend further studies to look into the preventive strategies already employed by patients to control their BP and assess their effectiveness.
